# AI-powered analysis of viral metagenomic sequencing data for rapid outbreak investigation and novel pathogen discovery

**DOI:** 10.3389/fmicb.2025.1717859

**Published:** 2026-01-12

**Authors:** David Chisompola, Emmanuel Luwaya, John Nzobokela, Phinnoty Mwansa, Martin Chakulya

**Affiliations:** School of Medicine and Health Sciences, Mulungushi University, Livingstone, Zambia

**Keywords:** artificial intelligence, outbreak investigation, pandemic preparedness, pathogen discovery, viral metagenomics

## Abstract

Emerging viral outbreaks continue to pose a persistent global health threat, underscoring the urgent need for a shift from reactive to proactive health security strategies. Viral metagenomic next-generation sequencing (mNGS) offers an unbiased, powerful approach to pathogen detection and discovery, yet its utility has been constrained by the computational complexity and slow turnaround time of data analysis during outbreak crises. The integration of artificial intelligence (AI) and mNGS is dismantling these barriers, enabling faster, more scalable outbreak response. This review synthesizes how AI-driven analytics are transforming mNGS applications, from genome assembly to sequence classification, using advanced architectures such as convolutional neural networks, recurrent neural networks, and transformers. Beyond accelerating workflows, AI’s capacity for pattern recognition outperforms traditional homology-based methods, facilitating the discovery of novel viral families and tracing hidden transmission chains through anomaly detection. Nonetheless, critical challenges remain, including limited training data, the interpretability of AI models, and resource-intensive computational demands that risk widening an “AI divide” in global health. We evaluate these obstacles and highlight forward-looking strategies, including federated learning for privacy-preserving data sharing and explainable AI for improving trust and biological insight. Looking ahead, we envision an “AI-first” paradigm for outbreak preparedness, anchored in integrated “Digital Immune Systems” for continuous, global-scale surveillance. By framing the synergy between mNGS and AI as a transformative leap, this review underscores its potential to strengthen resilience against future pandemics.

## 1 Introduction

Emerging and re-emerging viral pathogens continue to threaten global health security, as evidenced by outbreaks of Ebola virus, Zika virus, SARS-CoV-2, and, most recently, Mpox (Han et al., 2023). These events highlight how infectious diseases can trigger epidemics and pandemics that overwhelm healthcare systems and cause widespread societal and economic disruption (Peters et al., 2020; Bavinger et al., 2020). The frequency of such outbreaks, particularly those of zoonotic origin, is increasing, driven by factors including climate change, ecological disruption, and intensified global connectivity (Wilder-Smith, 2021). A critical determinant in mitigating the impact of these events is the speed of the public health response. The rapid and accurate identification of the causative pathogen is the essential first step for initiating effective containment, guiding therapeutic development, and deploying public health interventions (Yimer et al., 2024).

To identify pathogens, public health laboratories employ a variety of testing methods. Traditional assays include microscopy, culture-based analyses, and immunoassays that detect either pathogen antigens or host immune responses (Miller et al., 2013; Roux et al., 2021). While highly specific, these methods often require prior knowledge of the pathogen and can be slow. The adoption of nucleic acid amplification tests (NAATs), such as (Polymerase chain reaction) PCR, marked a significant advancement in speed and sensitivity but remains inherently targeted (Miller et al., 2013; Khan et al., 2024). Despite the availability of conventional testing approaches, many samples submitted to public health laboratories during outbreaks remain undiagnosed, leaving critical questions unanswered and exposing the limitations of standard diagnostic methods. This diagnostic gap has positioned metagenomic next-generation sequencing (mNGS) as a pivotal frontier for novel viral discovery.

Viral metagenomic next-generation sequencing, which enables the analysis of DNA and/or RNA from a sample (Roux et al., 2021), has emerged as a powerful tool for pathogen detection (Khan et al., 2024). By comprehensively interrogating nucleic acids in clinical and environmental samples, mNGS can identify known and novel viruses without prior knowledge of a causative agent (Roux et al., 2021; Khan et al., 2024; Mokili et al., 2012). This unbiased nature makes it indispensable for investigating unknown outbreaks. Despite its potential, the vast quantity and complexity of metagenomic datasets pose significant analytical challenges, especially when timely interpretation is critical during an emerging outbreak. However, analyzing mNGS data for novel viral discovery is challenging, as it demands specialized expertise in bioinformatics and data analysis. The introduction of artificial intelligence (AI) tools in mNGS is reshaping this landscape by enabling faster and more accurate interpretation of sequencing data, thereby accelerating the identification of novel pathogens.

Artificial intelligence such as, deep learning (DL) and machine learning (ML) have increasingly been recognized as transformative tools in biomedical data science, offering novel solutions for pattern recognition, anomaly detection, and predictive modeling (Hanna et al., 2025; Srivastava et al., 2025). In viral metagenomics, AI-powered methods have the potential to accelerate genome assembly, improve classification of unknown sequences, and uncover features that may otherwise be overlooked by conventional bioinformatics pipelines (Dhaarani and Reddy, 2025). The integration of AI into mNGS analysis promises not only to enhance outbreak investigations (Haug and Drazen, 2023), but also to expand the discovery of novel viruses with epidemic or pandemic potential (Liu, 2025).

Despite these advances, the application of AI in viral metagenomics is still in its early stages. Challenges such as data quality, model interpretability, computational resource demands, and ethical considerations hinder widespread adoption, particularly in resource-limited settings (Chong et al., 2025). Although AI has shown impressive advances in medicine, its application has faced significant obstacles especially when dealing with sensitive patient data. As global health systems seek faster and more reliable outbreak detection methods, understanding the opportunities and limitations of AI-driven metagenomic analysis becomes increasingly important.

While previous reviews have examined viral metagenomics and AI separately, few have explored their integration in outbreak investigation. This review provides a comprehensive overview of how the synergy between mNGS, and AI transforms rapid outbreak response and the systematic exploration of novel viruses. We outline current sequencing platforms, analytical frameworks, and AI applications for pathogen detection and discovery, while addressing key challenges, ethical considerations, and future directions. By consolidating recent evidence, we emphasize the potential of AI-powered metagenomics to advance outbreak investigation and novel pathogen discovery.

## 2 Methods for literature review

To ensure a comprehensive and systematic synthesis of current knowledge, this review was conducted according to a structured methodological framework.

### 2.1 Information sources and search strategy

A systematic literature search was performed across three major bibliographic databases: PubMed, Scopus, and Web of Science. To capture the most recent advancements, the search was limited to articles published between January 2014 and September 2025. The search strategy employed a combination of keywords and Medical Subject Headings (MeSH) terms related to the core concepts. The primary search string was: (“viral metagenomic*” OR “metagenomic next-generation sequencing” OR “mNGS”) AND (“artificial intelligence” OR “machine learning” OR “deep learning”) AND (“outbreak investigation” OR “pathogen discovery” OR “pandemic preparedness”). This string was adapted to the syntax requirements of each database.

### 2.2 Inclusion and exclusion criteria

The inclusion criteria prioritized peer-reviewed original research articles, case studies, reviews, and seminal methodological papers. Eligible studies were those published in English and focused on the application of AI/ML to mNGS data for viral detection, classification, or outbreak analytics. Studies addressing the use of mNGS in outbreak investigations or novel pathogen discovery were also included. However, studies were excluded if they focused exclusively on bacterial, fungal, or parasitic pathogens without a viral component. Articles were also excluded if AI/ML was applied solely to non-metagenomic data, such as medical imaging or electronic health records, without a direct connection to genomic sequence analysis. Conference abstracts, editorials, non-peer-reviewed commentaries, and studies lacking full-text availability were similarly omitted.

### 2.3 Study selection process

**Figure 1:**
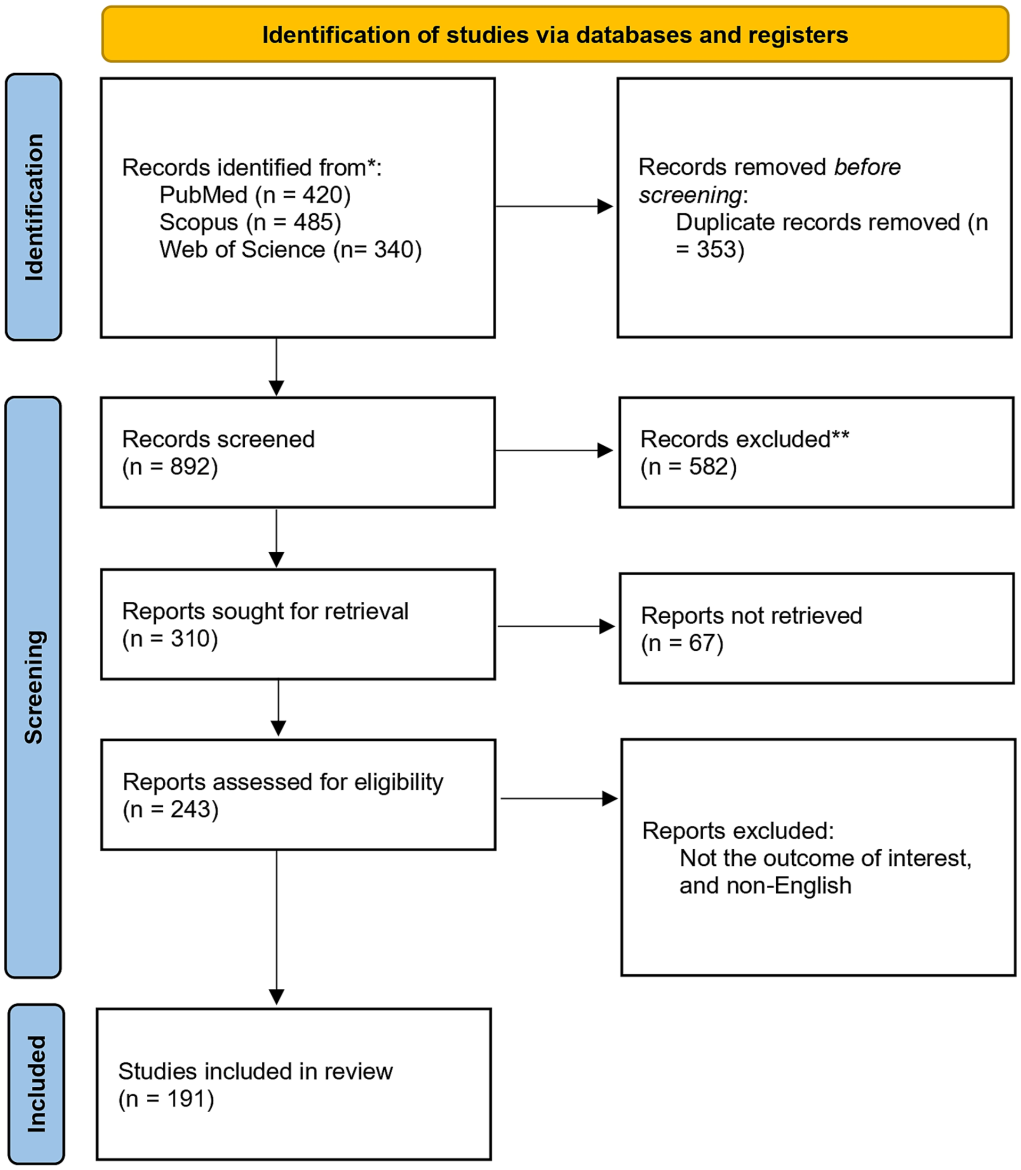
PRISMA flow diagram of literature selection.

The search results from all databases were consolidated, and duplicate records were removed using reference management Zotero version 7.0.30. The selection process adhered to a two-stage screening protocol to ensure rigor and minimize bias. First, two independent reviewers screened the titles and abstracts of all retrieved records against the predefined eligibility criteria. Any discrepancies or conflicts regarding inclusion at this stage were resolved through consensus discussion or, when necessary, arbitration by a third senior reviewer. Second, the full-text articles of all records deemed potentially relevant during the initial screening were retrieved and subjected to a comprehensive eligibility assessment by the same two reviewers. Final inclusion decisions were made based on strict application of the criteria. The results of this systematic selection process are detailed in the PRISMA-style flow diagram (Figure 1).

### 2.4 Data extraction and synthesis

Data from included studies were extracted into a standardized form, capturing information on study objectives, AI/ML methodologies, sequencing platforms, key findings, and identified challenges. Given the diverse and rapidly evolving nature of the field, a narrative synthesis approach was employed. Findings were thematically organized to construct a coherent overview of current applications, comparative advantages, persistent challenges, and future directions of AI-powered viral metagenomics.

## 3 An integrated framework for outbreak response

The rapid characterization of a viral pathogen is a critical determinant in the successful containment of an outbreak and the mitigation of its public health impact. No single technology operates in a vacuum; rather, a synergistic combination of tools creates a powerful, multi-layered defense system (Thomas et al., 2012). This integrated framework strategically leverages the unique strengths of rapid antigen tests, portable sequencing, high-throughput genomics, and artificial intelligence to create a cohesive pipeline from initial suspicion to definitive public health action (Linares et al., 2020).

Rapid antigen tests (RATs), serve as the crucial first line of defense (Cantón Cruz et al., 2025). These lateral flow immunoassays detect the presence of viral proteins, providing results in 15–30 min at the point of care. Their primary role is rapid case identification and triage, enabling immediate isolation and the prompt initiation of contact tracing to break chains of transmission in an outbreak’s early stages (Yimer et al., 2024). One of their key strengths is that RATs can be deployed without the need for specialized infrastructure, requiring only minimal tools, which has made them particularly valuable in resource-limited settings. However, their analytical performance, particularly sensitivity and specificity, has shown considerable variability, necessitating confirmation with PCR-based methods (Yimer et al., 2024; Hirabayashi et al., 2024).

PCR based techniques remain the gold standard for detecting viral infections, including SARS-CoV-2 (Cantón Cruz et al., 2025), and mpox in recent outbreaks (da Silva et al., 2023; Li et al., 2010). Despite their high analytical performance, particularly in terms of sensitivity, specificity, and throughput capacity for testing large volumes of suspected cases within a limited time, PCR methods are constrained by high costs, long turnaround times, reliance on skilled personnel, and potential exposure risks at testing sites. Nonetheless, PCR continues to be the preferred method at many facilities, while RATs are increasingly adopted as complementary tools in outbreak settings (Cantón Cruz et al., 2025).

During outbreaks, neither RATs nor PCR techniques have demonstrated the capacity to identify novel viral pathogens, as both depend on prior knowledge of existing viruses for their design and clinical utility. This limitation underscores the need for advanced sequencing technologies to enable novel pathogen discovery. Several sequencing platforms are available, including first-generation methods such as Sanger sequencing, second-generation platforms like Illumina, and third-generation technologies such as PacBio (Heather and Chain, 2016).

Sanger sequencing, also known as the chain-termination method, was developed by Frederick Sanger in 1977 and is considered the first-generation DNA sequencing technology (Eren et al., 2022). It relies on the selective incorporation of chain-terminating dideoxynucleotides (ddNTPs) during DNA synthesis, producing fragments of varying lengths that can be resolved by capillary electrophoresis to determine the nucleotide sequence (Heather and Chain, 2016; Eren et al., 2022). Although limited by its application in large-scale outbreak settings, relatively low throughput, short read lengths, and higher costs compared to next-generation sequencing (NGS) methods, Sanger sequencing remains widely used due to its high accuracy, reliability, and suitability for small-scale projects such as gene validation, clinical diagnostics, and confirmatory sequencing. Its precision in detecting single nucleotide variants continues to make it a valuable reference standard, even in the era of high-throughput sequencing technologies.

Illumina sequencing delivers high-fidelity data for definitive analysis. Typically deployed on PCR or RAT-positive samples, Illumina’s high accuracy and throughput are the cornerstone of genomic epidemiology (Huang et al., 2019). It enables precise reconstruction of transmission chains through whole-genome sequencing, distinguishes between multiple introductions of a virus, and powers large-scale surveillance to monitor for variants of concern. These attributes make Illumina particularly well-suited for outbreak investigations and the detection of novel viral pathogens.

Oxford Nanopore Technologies (ONT) sequencing provides real-time genomic intelligence. The portability of devices like the MinION allows for sequencing to be deployed directly in the field or in regional laboratories (Lu et al., 2016). This facilitates rapid initial characterization of an outbreak, enabling immediate detection of genetic drift or the emergence of a novel variant. Most significantly, its capacity for long-read, unbiased metagenomic sequencing makes it a powerful tool for *de novo* viral discovery when targeted tests are negative.

Sequencing platforms generate massive datasets that necessitate robust bioinformatic analysis. The sheer volume of data makes timely sequence interpretation challenging, and bioinformatic tools are essential for identifying novel viral pathogens. However, traditional bioinformatics is often constrained by the computational resources, costs, and specialized expertise required, limitations that are especially pronounced in resource-limited settings. Since the emergence of artificial intelligence (AI), there has been a growing effort to develop and validate automated AI-driven tools capable of analyzing sequencing data rapidly, enabling near real-time insights to support public health responses during outbreaks.

**Figure 2:**
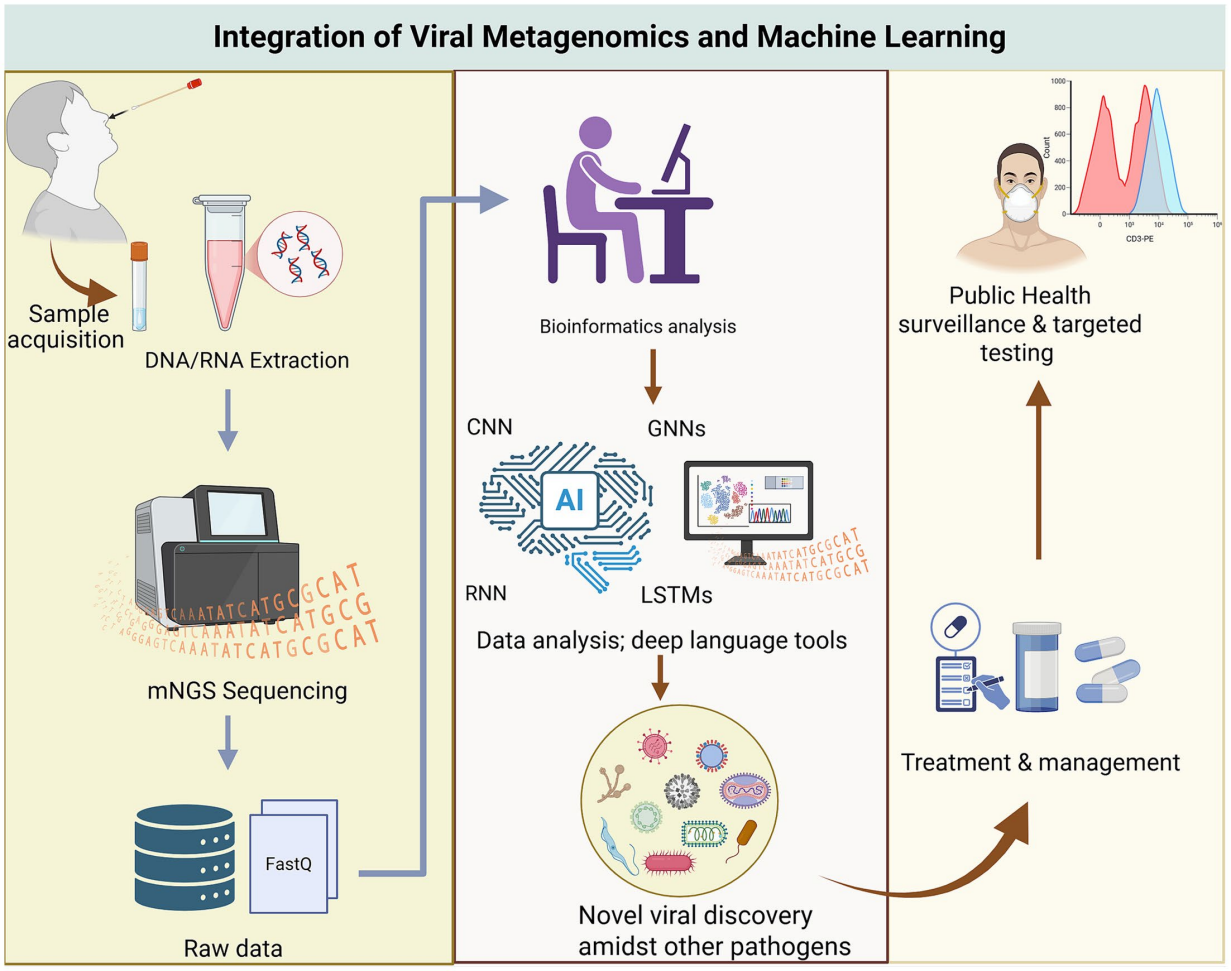
Integration of viral metagenomics with AI intelligence, machine learning and deep learning.

The full potential of this approach is realized through integration (Figure 2). In this framework, samples collected from humans, animals, or environmental sources are routed for sequencing. ONT provides rapid, near-source intelligence for initial outbreak characterization, while Illumina supplies high-fidelity data for definitive reconstruction and long-term surveillance. This combination of speed, portability, and accuracy forms a robust system for mitigating the impact of viral outbreaks and accelerating the discovery of emerging pathogens.

## 4 Traditional outbreak screening methods and their limitations

In the recent past, outbreak screening has facilitated the development and refinement of a wide range of diagnostic methods for the early detection of infectious diseases (Watkins et al., 2006). These include culture-based techniques, direct microscopy, immunoassays (antigen and antibody detection), and targeted NAATs such as PCR (Ieven, 2007). Traditional methods have been instrumental not only in guiding clinical diagnosis but also in paving the way for more advanced techniques. However, evidence suggests that there is considerable variability in their application during outbreak investigations, with no single method consistently preferred across different pathogens or public health settings. This inconsistency stems largely from differences in analytical performance, the availability of technical expertise, infrastructure demands, turnaround times, and associated costs.

Traditional outbreak screening methodologies have primarily relied on a combination of clinical suspicion and targeted laboratory testing (Abat et al., 2016). Symptoms alone are rarely sufficient for accurate identification, especially given that many pathogens produce overlapping clinical syndromes. As a result, clinicians, including physicians, veterinarians, nurse practitioners, and pathologists, play a central role in identifying suspected cases by screening patients presenting with compatible symptoms, collecting appropriate specimens, and initiating laboratory confirmation (Wagner et al., 2006). In this way, clinical suspicion provides a gateway for standardized case definitions and subsequent confirmatory laboratory testing. Conventional diagnostic tools such as real-time RT–PCR and viral culture have thus been central to outbreak detection (Reintjes and Zanuzdana, 2009). Nonetheless, despite their long history of use, these traditional approaches face significant challenges related to speed, accuracy, cost, and scalability in the context of modern outbreaks.

### 4.1 Direct microscopy

Direct microscopy, including electron microscopy (EM), remains a rapid tool for the presumptive identification of pathogens, allowing direct visualization of viral particles without prior knowledge (Apollon et al., 2022). Historically, EM played a decisive role in virus discovery, such as the 1948 differentiation of smallpox from chickenpox (Goldsmith and Miller, 2009). advances like transmission electron microscopy (TEM) and cryogenic electron microscopy (cryo-EM) (Richert-Pöggeler et al., 2019), sensitivity is generally lower than culture or PCR, and diagnostic error is a risk, as seen during coronavirus outbreaks (Bullock et al., 2022; Curry, 2003). Furthermore, in the case of SARS-CoV-2, EM confirmation of viral presence has been limited to select tissues (lung, heart, olfactory mucosa, and placenta), with inconsistent findings elsewhere (Birkhead et al., 2021). Such limitations highlight that, while EM remains invaluable for the discovery and confirmation of novel or unusual pathogens, it is not suitable for routine or large-scale outbreak diagnostics.

### 4.2 Polymerase chain reaction methods

The advent of nucleic acid amplification techniques, particularly PCR, transformed diagnostic virology beginning in the 1970s (Leland and Ginocchio, 2007). PCR enables sensitive and highly specific detection of viral nucleic acids without the need for viral propagation in culture, making it a faster and more versatile tool than traditional methods. Modern real-time PCR and multiplex NAAT platforms now allow simultaneous detection of up to 15 viruses and 4 bacteria in a single assay, representing a major advancement in outbreak screening, particularly for respiratory infections (Das et al., 2015).

PCR methods typically achieve sensitivity near 95% and specificity approaching 100%, which has established them as the gold standard in many diagnostic contexts. Their ability to detect low viral loads early in infection is particularly advantageous during outbreak investigations, where rapid case identification is critical. However, PCR is not without limitations. False positives may occur due to sample contamination, while false negatives may result from poor sample quality, inhibitors, or genetic mutations in the target region (Ieven, 2007; Iwata, 2020). Furthermore, high costs of reagents, consumables, and equipment, as well as the requirement for reliable electricity and trained staff, limit widespread accessibility in many resource-constrained settings. Despite these barriers, PCR remains the most widely adopted tool for outbreak detection, bridging the gap between clinical suspicion and definitive laboratory confirmation.

### 4.3 Culture-based techniques

For much of the 20th century, culture-based methods were the cornerstone of viral diagnostics and were long regarded as the gold standard for pathogen identification (Leland and Ginocchio, 2007). Viruses such as vaccinia, smallpox, and yellow fever were among the earliest to be propagated in culture between 1913 and the 1950s, with subsequent breakthroughs following the discovery that poliovirus could grow in non-neural cell lines. Viral culture remains unmatched in its ability to generate live isolates for further characterization, including drug susceptibility testing, antigenic typing, and vaccine development.

However, culture-based techniques are inherently slow, often requiring days to weeks to yield results, an unacceptable delay in the context of rapidly evolving outbreaks. They are also technically demanding, requiring specialized laboratory facilities, strict biosafety protocols, and highly trained personnel. Contamination risks further complicated interpretation, sometimes necessitating repeat cultures and extending diagnostic timelines. In many low- and middle-income countries, inadequate infrastructure and resource constraints have restricted the use of viral culture in routine outbreak surveillance.

While culture retains value for research, reference laboratories, and vaccine development, its role in frontline outbreak detection has largely been superseded by faster and more sensitive molecular methods.

### 4.4 Immunoassays (antigen and antibody detection)

Immunoassays remain a cornerstone in viral discovery due to their specificity, sensitivity, and relative ease of implementation (Wang et al., 2023). These assays leverage highly selective interactions between viral antigens and host antibodies or between antibodies and viral antigens, enabling both direct and indirect detection of viral pathogens. The two primary categories—antigen detection and antibody detection—serve complementary roles in uncovering known and novel viruses (Pavia and Plummer, 2021).

Antigen-based immunoassays detect viral proteins directly in clinical or environmental samples, providing evidence of active infection (Louten, 2016). Techniques such as enzyme-linked immunosorbent assays (ELISAs), lateral flow assays, and chemiluminescent immunoassays utilize monoclonal or polyclonal antibodies to capture and quantify viral antigens. In the context of viral discovery, these assays can rapidly screen large sample sets, flagging potential cases for more detailed molecular characterization. For example, during early outbreak investigations, antigen assays have been critical in identifying emerging influenza strains or novel coronaviruses (Cantón Cruz et al., 2025; Hirabayashi et al., 2024; Pavia and Plummer, 2021), often preceding nucleic acid-based confirmation.

Serological immunoassays detect host antibodies generated in response to viral infection, offering insights into exposure history and immune response dynamics. ELISA, Western blotting, and multiplex immunoassays allow high-throughput screening for IgM, IgG, or IgA antibodies against viral antigens. In viral discovery, antibody detection is particularly valuable for identifying past or subclinical infections, uncovering viruses that may evade direct detection (Louten, 2016). Serology can also guide epidemiological investigations, revealing the prevalence and distribution of previously unrecognized viral pathogens in populations.

Immunoassays are often used in tandem with molecular techniques to increase detection sensitivity and validate findings (Wang et al., 2023). For emerging viruses with limited genomic information, immunoassays can provide the first clues of viral presence by recognizing conserved structural proteins or cross-reactive epitopes. Furthermore, advances in recombinant antigen production, high-affinity antibody engineering, and multiplexed assay platforms have expanded the ability to detect multiple viral targets simultaneously, accelerating the pace of discovery (Matsunaga and Tsumoto, 2025).

Despite their utility, immunoassays face several limitations. Cross-reactivity with related viruses may produce false positives, particularly in antibody-based assays (Luvira et al., 2022). Antigen assays may have reduced sensitivity in low-viral-load samples, while serology is limited by the window period between infection and detectable antibody production. Nevertheless, when carefully designed and interpreted in conjunction with complementary techniques such as metagenomic sequencing, immunoassays provide a rapid, cost-effective, and scalable approach for identifying novel viruses and monitoring emerging infectious threats.

### 4.5 DNA microarray

DNA microarray technology emerged as an advanced diagnostic tool for infectious diseases, designed to enable the simultaneous and specific detection of a wide range of pathogens (Asmare and Erkihun, 2023). The principle of detection relies on solid-phase hybridization, where pathogen-specific oligonucleotide probes are immobilized on a solid surface and hybridize with complementary sequences from a mixture of fluorescently labeled nucleic acids (Martínez et al., 2014). Over time, diverse microarray platforms were developed to target pathogens associated with respiratory, hemorrhagic, blood-borne, and central nervous system syndromes (Martínez et al., 2014), while broader-spectrum microarrays were designed for virus discovery and surveillance (Wang et al., 2002).

This technology represented a pivotal step forward in molecular diagnostics, as it enabled the parallel screening of thousands of predefined viral sequences on a single chip through probe–target hybridization (Wang et al., 2003). Compared with single-plex PCR, microarrays offered a much wider scope of detection. However, they remained fundamentally targeted: probe design required prior knowledge of pathogen genomic sequences, restricting their ability to identify novel or highly divergent agents. Additional challenges included cross-hybridization artifacts, which could compromise specificity, and a generally lower sensitivity compared to amplification-based methods.

As a result, while DNA microarrays played an important transitional role in broad-spectrum pathogen detection, they were eventually surpassed in pathogen discovery by metagenomic next-generation sequencing. Unlike microarrays, metagenomic next-generation sequencing provides a truly unbiased approach, capable of identifying both known and previously uncharacterized pathogens, making it the focus of current and future innovations in infectious disease diagnostics.

## 5 Viral metagenomic next-generation sequencing

Viral metagenomics next-generation sequencing provides a fast, sensitive, and robust approach for detecting viruses, including those that remain undetectable by traditional culture techniques and sequence-dependent assays (Mokili et al., 2012). This unique capability has firmly established mNGS as a leading tool in the discovery of novel viruses. Unlike conventional diagnostic assays, which rely on prior knowledge of target sequences, mNGS employs an unbiased strategy that enables the simultaneous detection of both known and unknown viral pathogens. This makes it invaluable in situations where the causative agent of an outbreak is unknown, representing a critical first step in mounting an effective and timely outbreak response (Greninger, 2018). When both culture-based methods and advanced molecular assays fail to detect a pathogen, mNGS has often served as the ultimate diagnostic approach, leading to the identification of novel viruses (Figure 2; Roux et al., 2021).

Since the first viral genome was sequenced using metagenomic methods in 2002, the pace of virus discovery has accelerated dramatically (Mokili et al., 2012; Dutilh et al., 2017). A landmark example is the identification of SARS-CoV-2 in 2019, where mNGS enabled rapid characterization of the novel coronavirus and provided genomic data that informed early diagnostic test development, epidemiological modeling, and vaccine design. Two decades later, mNGS remains at the forefront of virology, now enhanced by the integration of artificial intelligence (AI), machine learning (ML), and deep learning tools, which increase the speed, accuracy, and interpretability of vast sequencing datasets.

The strength of mNGS lies in its flexibility and broad applicability. Unlike targeted assays such as PCR, which require specific primers, mNGS can be applied to a wide variety of sample types, including blood, respiratory swabs, stool, plant tissues, and environmental reservoirs such as wastewater, while still generating high-quality sequence data (Roux et al., 2021). This versatility is particularly significant in today’s context of frequent human-animal-environment interactions, where zoonotic spillover events have led to the emergence of high-impact pathogens such as Ebola virus, mpox virus, and coronaviruses. Beyond pathogen discovery, mNGS has proven valuable in detecting co-infections, characterizing viral diversity within hosts, and monitoring viral evolution. For example, it has successfully identified viral-bacterial co-infections such as varicella zoster virus with herpes simplex virus-2 (Schuele et al., 2025). Similarly, Slavov, reported that clinically important viruses, including measles virus, SARS-CoV-2, hepatitis B virus, parvovirus B19, adenovirus, and human herpesviruses, were detected alongside commensal members of the blood virome such as anelloviruses (Slavov, 2025). These findings highlight mNGS’s ability not only to detect pathogens but also to provide insights into the broader viral ecosystem associated with human health and disease.

The workflow of mNGS begins with specimen collection, which can include clinical samples (e.g., blood, cerebrospinal fluid, nasopharyngeal swabs), animal reservoirs, or environmental sources (e.g., soil and water). Viral nucleic acids (DNA and/or RNA) are then extracted, followed by random or targeted amplification and library preparation (Morgan et al., 2010). Sequencing is performed using high-throughput platforms such as Illumina, Oxford Nanopore Technologies, or Pacific Biosciences (PacBio), each offering distinct advantages in terms of read length, throughput, and error profile. The raw sequence data undergoes comprehensive bioinformatics processing, including quality filtering, host read subtraction, *de novo* assembly, and taxonomic classification, to achieve viral identification and characterization (Slavov, 2025; Alcolea-Medina et al., 2024; Chiu and Miller, 2019).

Despite its transformative power, the application of mNGS requires specialized expertise, sophisticated equipment, significant computational resources, and robust laboratory and bioinformatics infrastructure. These requirements currently limit their widespread use in low- and middle-income countries where outbreak risks are often highest. Nevertheless, the unbiased nature of mNGS makes it uniquely suited for outbreak investigations and pathogen surveillance. Its real-world impact has been repeatedly demonstrated, such as in the rapid identification of SARS-CoV-2 in Wuhan in 2019, the genomic characterization of mpox outbreaks, and the elucidation of viral genomes during Zika and Ebola epidemics. Moving forward, continued improvements in sequencing technology, data analysis pipelines, and cost reduction, alongside integration with AI-driven analytics, will further strengthen the role of mNGS in global health security and pandemic preparedness.

### 5.1 Metagenomic next generation sequencing technologies

Metagenomic next-generation sequencing platforms are now widely employed not only for targeted sequencing of specific genes or genomic regions but also for comprehensive, sequence-based association analyses that drive pathogen discovery and characterization (Harismendy et al., 2009). Their growing adoption is largely fueled by the urgent need for faster, more accurate, and versatile diagnostic tools in infectious disease management. Beyond diagnostics, mNGS has opened entirely new avenues for research by allowing scientists to interrogate genetic information at an unprecedented scale and resolution, thereby advancing our understanding of microbial diversity, host–pathogen interactions, and evolutionary dynamics (Goodwin et al., 2016).

Technology’s integration of high-throughput performance with steadily improving affordability has solidified its role as a cornerstone in fields spanning from fundamental biology and epidemiology to precision medicine and clinical diagnostics. While debates persist regarding their overall cost-effectiveness, particularly in low-resource settings, the practical utility of mNGS in accelerating discovery and improving diagnostic sensitivity has proven invaluable. Importantly, one of its defining advantages lies in its ability to generate vast volumes of sequence data, typically ranging from 300 to 500 cycles per run, enabling deep coverage and comprehensive genomic profiling (Kozich et al., 2013).

Nevertheless, sequencing performance varies considerably across platforms, with notable differences in read length, accuracy, throughput, and error profiles that directly impact downstream analyses and clinical applications. Some technologies are optimized for generating short, highly accurate reads, whereas others prioritize long-read sequencing, which is advantageous for genome assembly and structural variant detection. Historically, Illumina’s short-read (second-generation) sequencing has dominated mNGS because of its high accuracy and throughput. In contrast, the advent of third-generation platforms such as Oxford Nanopore Technologies (ONT) and Pacific Biosciences (PacBio) has revolutionized the field by enabling long-read sequencing, often spanning thousands of bases, with steadily improving accuracy (Han et al., 2024). While short reads (<300 bp) can lead to fragmented assemblies and may overlook structural variants, long reads provide the ability to resolve repetitive regions and capture full-length genes, thereby offering significant advantages in metagenomic applications (Han et al., 2024). Below we compare Illumina, ONT, and PacBio across key metrics for metagenomics (Table 1).

**Table 1:** Comparison of major high-throughput sequencing platforms for viral metagenomics.

Feature	Illumina (short-read)	Oxford nanopore technologies (ONT) (long-read)	Pacific biosciences (PacBio) (long-read, HiFi)
Key technology	Sequencing-by-synthesis with reversible dye-terminators (Chen et al., 2013).	Nanopore-based electronic sensing of DNA/RNA strands (Ni et al., 2019).	Single Molecule, Real-Time (SMRT) sequencing with circular consensus sequencing (CCS) (Zhang et al., 2025).
Typical read length	50–300 bp (short) (Kozich et al., 2013).	10 kb–100+ kb (ultra-long) (Moustakli et al., 2025; Shafin et al., 2020).	Long & Accurate (15–25 kb) (HiFi reads) (Wen and Tang, 2025; Travers et al., 2010; Wenger et al., 2019).
Throughput and speed	Very High throughput (Terabases/run). Run time: hours to days (Liu et al., 2021).	Scalable & Real-Time. From portable (MinION) to high throughput (PromethION). Data is available immediately. Scalable (MinION: ~30 Gb; PromethION: ~13 Tb) (Chen and Xu, 2023).	High (Revio: ~100–120 Gb per SMRT Cell) (Zhang et al., 2025).
Accuracy profile	Very High (>99.9%); substitution errors (Schirmer et al., 2016).	Moderate-High (Raw: ~99%; with Q20+: >99.9%); indel errors in homopolymers (Sereika et al., 2022; Chen et al., 2024).	Extremely High (>99.9% with HiFi mode) (Wen and Tang, 2025; Travers et al., 2010; Wenger et al., 2019).
Primary strengths	Gold standard for cost-effective, high-depth sequencing. Excellent for SNP/variant calling and quantitative abundance (Xia et al., 2023; Logares et al., 2014).	Real-time analysis for immediate insights. Extreme portability for field use. Best for detecting structural variants and *de novo* assembly. Direct RNA sequencing (De Coster et al., 2019).	Unparalleled combination of long reads and high accuracy. Ideal for resolving complex regions, haplotype phasing, and high-quality de novo assembly (Slavov, 2025; Alcolea-Medina et al., 2024; Chiu and Miller, 2019).
Primary limitations	Short reads fail to resolve repeats, leading to fragmented assemblies. Cannot phase haplotypes or detect large Structural Variants s easily (González et al., 2025).	Historically higher error rate, though improving rapidly. Requires substantial bioinformatics for basecalling and error correction (Sereika et al., 2022; Chen et al., 2024).	Highest per-sample cost. Requires high-quality, high molecular weight DNA input. Less suitable for rapid, real-time applications (Han et al., 2024).
Best suited for mNGS application	Large-scale surveillance and detection: Sensitive pathogen identification in complex samples (e.g., microbiome, plasma). High-resolution SNP analysis for outbreak tracing.(Xia et al., 2023).	Rapid Real-time pathogen detection and outbreak response in the field. *De novo* assembly of unknown pathogens. Epigenetic modification detection (Marić et al., 2024).	Gold-standard de novo genome assembly of novel viruses/bacteria. Resolving complex viral communities and strain variants. Full-length 16S/18S sequencing without amplification bias (Slavov, 2025; Alcolea-Medina et al., 2024; Chiu and Miller, 2019).

#### 5.1.1 Illumina sequencing platforms

Illumina-based sequencing technology is one of the most widely adopted platforms in metagenomics, providing high-throughput and cost-effective sequencing of DNA and RNA (Xia et al., 2023; Schirmer et al., 2016). It has become a cornerstone for microbial community analysis, particularly for taxonomic profiling, functional annotation, and pathogen detection (Elbehiry and Abalkhail, 2025). The platform’s short-read approach delivers massive sequencing depth at relatively low cost, making it ideal for large-scale studies across clinical, environmental, and engineered systems (Xia et al., 2023). Illumina short reads enable sensitive and robust detection of microbial community composition, antimicrobial resistance genes, and metabolic pathways, provided sufficient coverage is achieved. Furthermore, metagenomic Illumina tags (_mi_Tags) can mitigate PCR amplification biases, offering more accurate estimates of microbial richness and evenness compared with traditional amplicon-based methods (Logares et al., 2014). Nonetheless, with modern instruments such as the NovaSeq 6000 and NovaSeq X series, Illumina can yield terabase-scale data in a single run, supporting large-scale environmental surveys and deep sequencing for rare pathogen detection.

Despite these strengths, Illumina sequencing introduces several challenges and biases. Position- and motif-specific errors, most commonly substitution errors linked to specific nucleotide motifs, may persist even after quality filtering and affect downstream analyses (Schirmer et al., 2016). The reliance on short reads (<300 bp) can lead to fragmented assemblies, particularly in complex communities or genomes with high sequence similarity, thereby limiting recovery of complete genomes and structural variants (González et al., 2025). Low-abundance organisms or genes may also be difficult to detect without very deep sequencing (e.g., ~30 million reads for 1% abundance) (Rooney et al., 2022).

To overcome these limitations, hybrid strategies that combine Illumina with long-read platforms such as Oxford Nanopore Technologies (ONT) or Pacific Biosciences (PacBio) are increasingly employed (Xia et al., 2023; Sevim et al., 2019). These approaches improve assembly contiguity, genome completeness, and the resolution of structural variants, while retaining Illumina’s advantage in low error rates and reliable genome recovery. Ultimately, Illumina remains foundational in metagenomics for its accuracy, throughput, and affordability, but optimal study design requires careful consideration of its error profiles and assembly constraints. In many cases, hybrid or long-read approaches provide a more comprehensive view of microbial diversity and genome structure.

#### 5.1.2 Oxford nanopore technologies

Oxford Nanopore Technologies (ONT) has transformed metagenomics by enabling real-time, portable, and long-read sequencing of complex microbial communities (Ni et al., 2019). ONT devices employ protein nanopores embedded in membranes to directly read native DNA or RNA molecules as they translocate through the pore, generating reads of virtually unlimited length, routinely exceeding 100 kilobases (kb) and occasionally surpassing 1 megabase (Mb) (Moustakli et al., 2025; Shafin et al., 2020). This ability to generate ultra-long reads is ONT’s defining feature, enabling more contiguous genome assemblies, improved detection of structural variations, and strain-level resolution in metagenomic samples. Compared with short-read platforms, ONT excels at resolving repetitive regions and reconstructing complex genomes.

ONT platforms, including the portable MinION and high-throughput PromethION, are increasingly applied across clinical diagnostics, outbreak investigations, environmental monitoring, and food safety (De Coster et al., 2019). Technology is particularly valuable for rapid pathogen detection, often providing actionable results within hours. For example, ONT sequencing has been deployed in real-time outbreak response and field-based surveillance due to its portability and minimal infrastructure requirements (Oehler et al., 2023). Output scales from ∼30–50 Gb on a MinION flow cell to up to ∼13 Tb on a PromethION run (48 flow cells, 72 h), making it suitable for both targeted and large-scale applications (Chen and Xu, 2023).

Despite these advantages, ONT historically faced limitations due to higher raw error rates (5–10%), predominantly indels in homopolymeric regions. Although consensus polishing with short reads was often required, recent advances, including R10.4.1 nanopores and Q20 + chemistry—have markedly improved basecalling performance, achieving raw-read accuracies exceeding 99% (Sereika et al., 2022; Chen et al., 2024). Nevertheless, homopolymer-associated errors remain a challenge, and robust bioinformatics workflows are required for error correction, host DNA contamination filtering, and metagenome-assembled genome (MAG) reconstruction. Hybrid approaches that integrate ONT with Illumina sequencing remain the gold standard for producing highly complete and accurate assemblies.

Specialized tools (e.g., Pike for OTU-level analysis) and the development of field-adapted extraction protocols continue to expand ONT’s utility, offering flexible and cost-effective solutions for microbial surveillance and biodiversity studies (Krivonos et al., 2025). Overall, ONT’s key strength lies in its real-time sequencing capability, enabling rapid clinical diagnostics, novel pathogen surveillance, and metagenomic assemblies requiring ultra-long reads. However, it’s per-base cost remains higher than Illumina for very large-scale projects, and careful consideration of study goals is necessary when selecting ONT as a primary sequencing platform.

#### 5.1.3 Pacific biosciences (PacBio)

Pacific Biosciences has established itself as the leader in high-fidelity long-read sequencing through its circular consensus sequencing (CCS) strategy, yielding HiFi reads that combine long-read lengths (typically 15–25 kb) with accuracies exceeding 99.9% (Q30 or higher) (Wen and Tang, 2025; Travers et al., 2010; Wenger et al., 2019). The latest Revio platform generates ~100–120 Gb per SMRT Cell, with up to four cells running in parallel, enabling hundreds of gigabases of highly accurate long-read data per day (Zhang et al., 2025). PacBio’s requirement for high molecular weight DNA and relatively complex library preparation workflows pose technical challenges, but the resulting data are uniquely powerful. HiFi reads preserve single-nucleotide accuracy while spanning long genomic regions, enabling recovery of complete metagenome-assembled genomes (MAGs), structural variant resolution, and improved taxonomic resolution for rare or novel taxa. Comparative studies have shown that PacBio recovers more low-abundance lineages than Illumina or ONT, due to its combination of read length and accuracy. The high capital cost of PacBio systems and consumables may restrict adoption in resource-limited settings, but for high-resolution metagenomics, especially in research on complex microbial communities, PacBio HiFi data are considered the gold standard.

## 6 Current approaches to data analysis

Traditional alignment-assembly-annotation pipelines remain the backbone of viral metagenomic sequencing for outbreak investigation (Yang et al., 2024). They provide interpretable and clinically actionable results: read classification enables rapid confirmation of suspected pathogens, genome assemblies allow high-resolution phylogenetic analyses, and annotation facilitates detection of resistance or virulence markers (Song et al., 2021; Rosenboom et al., 2022). Benchmarking studies of clinical metagenomic pipelines confirm that these workflows are highly specific for known pathogens and reproducible across laboratories, supporting their integration into surveillance and public health responses (de Vries et al., 2021). The assembled genomes were central in tracking SARS-CoV-2 lineage dynamics during the COVID-19 pandemic, enabling timely insights into transmission, mutation hotspots, and global spread (Saravanan et al., 2022). The maturity of these pipelines, coupled with standardized platforms such as Nextflow and Snakemake, ensures reproducibility and traceability, critical strengths during time-sensitive outbreak responses (Langer et al., 2025).

On the other hand, recent studies confirm important limitations when applying traditional pipelines for outbreak investigation and novel pathogen discovery. Wet-lab protocol comparisons have demonstrated that enrichment via capture panels dramatically increases sensitivity in low viral load samples: for respiratory pathogens such as SARS-CoV-2 or influenza A, target capture sequencing yielded 180–2,000-fold higher viral read counts compared to untargeted metagenomics in some clinical specimens (Takemae et al., 2024). Benchmarking of virus identification tools using real-world metagenomic datasets found that while some tools perform well under default settings, there is a trade-off between sensitivity and specificity and many tools fail to recover virus contigs when genomes are fragmented or diverged (Takemae et al., 2024). Therefore, reliance solely on traditional pipelines can delay detection of novel or low-abundance pathogens in outbreak settings, unless supplemented with optimized sample preparation, high sequencing depth, and tools designed to handle divergent sequences (Wu et al., 2024).

## 7 AI and machine learning applications

The rapid expansion of metagenomic sequencing has generated unprecedented volumes of complex and heterogeneous data, necessitating advanced analytical frameworks beyond conventional bioinformatics (Pita-Galeana et al., 2025). AI, particularly ML and DL, has emerged as a transformative tool for extracting biologically meaningful insights from metagenomic datasets. Its applications span the entire analytical pipeline, from raw data preprocessing to functional inference and clinical translation.

One of the central challenges in viral metagenomics is accurate classification of sequences, especially when viral genomes exhibit high mutation rates or when reference databases are incomplete. Traditional bioinformatics pipelines rely on alignment-based methods (e.g., BLAST, Bowtie) or k-mer frequency approaches (Pita-Galeana et al., 2025; Wu et al., 2021). These approaches are limited when viral sequences diverge significantly from known references.

Deep learning algorithms, particularly convolutional neural networks (CNNs) (de Souza et al., 2023), and recurrent neural networks (RNNs) (Deif et al., 2021), have been developed to overcome these limitations. By learning hierarchical sequence features directly from raw data, deep learning models can classify viral sequences with higher accuracy and generalize better to novel or divergent genomes. For instance, CNN-based models have been applied to detect viral families from short sequencing reads without requiring genome assembly (Tampuu et al., 2019).

Autoencoders and attention-based models (e.g., transformers) further extend classification performance by capturing long-range dependencies and sequence motifs relevant to viral taxonomy (Mswahili and Jeong, 2024). Importantly, these models can recognize “novelty signatures,” allowing for classification at higher taxonomic ranks when species-level resolution is not possible (Table 2). This feature is critical in outbreak investigations where the pathogen may belong to an underrepresented or previously unknown viral group.

**Table 2:** Key artificial intelligence architectures and their applications in viral metagenomics.

Architecture	Core mechanism	Key strengths in viral metagenomics	Primary applications and examples
Convolutional neural networks (CNNs)	Applies learnable filters to detect local, position-invariant patterns (Unsal et al., 2022; Bileschi et al., 2022).	Excellent motif discovery; robust to sequence variability; does not require high sequence homology (Ren et al., 2020; Ren et al., 2020).	Viral sequence classification: Distinguishing viral from host sequences (e.g., DeepVirFinder) (Ren et al., 2020; Ren et al., 2020). Family-level identification: Classifying unknown viruses based on conserved protein motifs (e.g., polymerase) (Mswahili and Jeong, 2024).
Recurrent neural networks (RNNs)/Long short-term memory (LSTMs)	Processes sequences step-by-step, maintaining a “memory” of previous context via hidden states (Nayfach et al., 2021).	Models short-to-long-range dependencies; captures sequential context and temporal dynamics (Bengio et al., 1994).	Modeling viral evolution: Tracking intra-host evolution and quasi-species dynamics during prolonged outbreaks (Singh et al., 2016). Functional annotation: Identifying gene boundaries (start/stop codons) and regulatory elements (Pasolli et al., 2019).
Transformers and attention mechanisms	Uses self-attention to weigh the importance of all positions in a sequence simultaneously (Bigness et al., 2022; Choi and Lee, 2023).	Captures long-range, global dependencies efficiently; enables parallel processing; highly adaptable via pre-training (Bigness et al., 2022; Choi and Lee, 2023).	*De novo* genome assembly: Improved assembly of novel viral genomes from complex metagenomic data (Slavov, 2025; Alcolea-Medina et al., 2024; Chiu and Miller, 2019). Host prediction and pathogenicity: Learning generalizable representations for tasks like host prediction (e.g., ViralBERT, PathogenTransformer) (Abràmoff et al., 2023; Vashisht et al., 2023; Choi and Lee, 2023).
Graph neural networks (GNNs)	Operates on graph structures where nodes (e.g., individuals, variants) are connected by edges (e.g., transmissions) (Bileschi et al., 2022; Liu et al., 2020).	Models relational and network data; integrates heterogeneous data types (genomic, mobility, contact) (Nasir et al., 2023).	Transmission dynamics: Reconstructing transmission chains and identifying superspreader events (Choi et al., 2022). Variant spread tracking: Integrating genomic and mobility data to project geographic spread of variants (Choi et al., 2022).
Autoencoders (for anomaly detection)	Compresses input data into a latent space and reconstructs it; high reconstruction error indicates anomalies (Marić et al., 2024; Zhao et al., 2023).	Unsupervised learning; does not require labeled data for novel pathogens; identifies deviations from known sequences (Barredo Arrieta et al., 2020).	Novel pathogen discovery: Flagging unclassified or highly divergent genomic fragments as potential novel viruses (Barredo Arrieta et al., 2020). Outbreak early warning: Detecting anomalous sequence clusters in surveillance data (Kuo and Ying, 2023).

### 7.1 Key architectures and applications

#### 7.1.1 Convolutional neural networks (CNNs)

Convolutional neural networks, initially developed for computer vision, have proven highly effective in biological sequence analysis due to their ability to detect local, position-invariant patterns, a defining property of genomic data (Rives et al., 2021; Rives et al., 2021). By applying learnable filters across nucleotide or amino acid sequences, CNNs act as motif discovery engines, identifying conserved short patterns such as transcription factor binding sites or protease cleavage motifs (Alley et al., 2019; Jumper et al., 2021; Senior et al., 2020). Sequences are typically represented numerically through one-hot encoding, where nucleotides or amino acids are mapped into binary vector space, forming input matrices analogous to images (Jumper et al., 2021). Convolutional and pooling layers then generate feature maps that summarize motif occurrence and position, conferring robustness to sequence variability (Unsal et al., 2022; Bileschi et al., 2022).

This hierarchical feature extraction enables CNNs to detect both simple motifs and complex higher-order structures, such as protein domains or viral polymerases (Hie et al., 2021; Ming et al., 2023; Lee, 2023). Consequently, CNNs can classify viral from non-viral sequences, annotate regulatory regions, and identify taxonomic signatures, even in cases of low sequence similarity to reference genomes (104). This capability to detect motifs without high-sequence homology is particularly advantageous over alignment-based methods like BLAST during the investigation of a novel outbreak. For instance, a CNN can enable family-level classification of an unknown virus based on conserved polymerase motifs, providing a crucial first clue for public health responders within hours, even when the virus shares less than 50% sequence similarity to any known reference. Tools like DeepVirFinder leverage CNN architectures to uncover viral sequences in metagenomic assemblies by integrating k-mer compositions with contextual genomic signals, outperforming alignment-based methods in novel virus discovery (Ren et al., 2020; Ren et al., 2020). As sensitive pattern detectors, CNNs thus provide a scalable solution for viral genome annotation and pathogen discovery in metagenomics.

#### 7.1.2 Recurrent neural networks (RNNs)

Recurrent Neural Networks are designed specifically for sequential data and thus provide a natural framework for nucleotide and amino acid sequence analysis (Chandra et al., 2023). Unlike CNNs, which specialize in local motif detection, RNNs capture dependencies across positions by processing sequences element-by-element while maintaining a hidden state that reflects prior context (Graves, 2012). This allows the model to incorporate the meaning of a nucleotide or codon in relation to its surrounding sequence, which is critical for recognizing reading frames, splice sites, and regulatory elements spanning long genomic distances (Rumelhart et al., 1986; Auslander et al., 2021).

However, conventional RNNs are hindered by the vanishing gradient problem, limiting their ability to learn long-range dependencies (Bengio et al., 1994). Despite this, they remain valuable for tasks requiring short- to medium-range contextual modeling, including identifying conserved sequence patterns, functional annotation of genes, and detecting short-range evolutionary constraints.

#### 7.1.3 Long short-term memory networks (LSTMs)

Long short-term memory networks extend RNNs by incorporating gating mechanisms, input, forget, and output gates, that regulate the retention and flow of information (Gers et al., 2000). This architecture mitigates vanishing gradients, enabling learning across long genomic regions where distant interactions carry biological significance.

In viral metagenomics, LSTMs are particularly effective in modeling genome-wide signals such as codon usage bias, oligonucleotide frequencies, and co-evolutionary patterns between viruses and hosts (Nayfach et al., 2021). They also excel at identifying start/stop codons, splice sites, and functional domains separated by introns or long intergenic regions (Pasolli et al., 2019). This is critical for modeling viral evolution within a host during a prolonged outbreak, such as an Ebola or SARS-CoV-2 epidemic, allowing researchers to track the emergence of quasi-species that may impact transmission or treatment efficacy. For phylogenetic classification, LSTMs capture patterns of mutation and conservation across entire genomes, producing robust evolutionary inferences beyond local homology (Singh et al., 2016). Although newer architectures such as Transformers (Tampuu et al., 2019), offer advantages in handling very long sequences with parallelization, LSTMs remain widely used due to their strong performance in tasks where sequential order and contextual dependencies are biologically essential (Jurtz et al., 2017).

### 7.2 Transformers and attention mechanisms

Transformers mark a major advancement in sequence analysis, diverging from convolutional and recurrent architectures through their core innovation: the self-attention mechanism (Choi et al., 2022). Unlike CNNs, which emphasize local motifs, or RNNs, which process sequences sequentially, transformers assign weights to all positions simultaneously, enabling direct modeling of long-range dependencies (Bigness et al., 2022; Choi and Lee, 2023). This “global receptive field” allows the model to capture distant but functionally linked genomic features, such as promoter–coding region interactions or co-evolutionary signals across protein domains (Marić et al., 2024; Zhao et al., 2023).

The multi-head attention framework further enhances representational capacity by attending to different subspaces in parallel, capturing both syntactic (e.g., reading frames) and semantic (e.g., protein domain function) dimensions of genomic sequences (Marić et al., 2024). By modeling entire genomes holistically, transformers facilitate more accurate *de novo* assembly of novel viral genomes from complex metagenomic samples, directly addressing the challenge of fragmented assemblies that can delay the development of confirmatory PCR tests. Pre-trained models such as ViralBERT and PathogenTransformer leverage massive viral sequence corpora to learn generalizable representations that can be fine-tuned for specific tasks, including host prediction, gene annotation, and pathogenicity classification (Abràmoff et al., 2023; Vashisht et al., 2023; Choi and Lee, 2023). More recent architectures, such as MetaViT, extend transformer applications to metagenomics, effectively identifying novel viral sequences by recognizing global genomic signatures beyond local homology (Ji et al., 2021). By modeling genomes holistically, transformers advance viral genomics toward context-aware interpretation and accelerate novel virus discovery.

### 7.3 AI in anomaly and outlier detection

Early identification of novel pathogens is a critical application of AI in outbreak surveillance (Jurtz et al., 2017). Traditional approaches, which rely on known genetic signatures or symptom patterns, are limited in detecting truly novel threats (Tisza and Buck, 2021). In contrast, unsupervised and semi-supervised learning models excel at anomaly detection by learning baseline distributions of genomic or clinical data and flagging deviations (Barredo Arrieta et al., 2020). Within metagenomic sequencing datasets, algorithms such as isolation forests and autoencoders can detect unclassified genomic fragments as potential novel viruses (Kuo and Ying, 2023). For example, high reconstruction error in autoencoders indicates sequences that diverge from known distributions, serving as a quantifiable anomaly score (Marić et al., 2024; Zhao et al., 2023).

Beyond genomics, AI pipelines integrate clinical and epidemiological metadata, such as geographic location, travel history, and symptom onset, with sequence anomalies to detect clusters of unexplained infections (Edwards et al., 2016). This proactive detection framework can generate early outbreak warnings well before traditional confirmation methods, potentially reducing response delays by weeks (Willmington et al., 2022; Rudin, 2019).

### 7.4 Predictive models for transmission dynamics

Following pathogen identification, AI-driven models enhance prediction of transmission dynamics, informing timely public health interventions (Lee et al., 2023). Traditional SEIR (Susceptible-Exposed-Infectious-Recovered) frameworks provide a foundation but rely on static parameters (Auslander et al., 2021; Cheohen et al., 2025). AI augments these models by integrating real-time data and adapting parameters dynamically. Time-series methods, particularly LSTMs, can incorporate case counts, human mobility, climate data, and social media signals to forecast short-term epidemic trends with greater accuracy (Choi and Lee, 2023; Kuo and Ying, 2023).

Graph neural networks (GNNs) extend predictive power by modeling transmission chains, representing individuals or communities as nodes and their interactions as edges (Bileschi et al., 2022; Liu et al., 2020). Such models can identify superspreader events, transmission hubs, and potential intervention points. Moreover, by incorporating genomic data, GNNs can track pathogen evolution alongside mobility-driven spread, enabling projections of both geographic expansion and variant dominance (Nasir et al., 2023). These integrative models support resource prioritization and targeted containment strategies, bridging epidemiological forecasting with genomic surveillance.

### 7.5 Case studies

#### 7.5.1 SARS-CoV-2

The COVID-19 pandemic served as a large-scale proving ground for AI in virology (Rives et al., 2021; Shkoporov et al., 2022). Deep learning models, most notably AlphaFold2, accurately predicted the 3D structure of the SARS-CoV-2 spike protein, which significantly accelerated rational vaccine design and therapeutic development (Jumper et al., 2021). AI-driven genomic surveillance systems played a crucial role in monitoring viral evolution by classifying variants of concern (e.g., Alpha, Delta, Omicron) through detection of mutational signatures associated with increased transmissibility, pathogenicity, and immune escape In parallel, natural language processing (NLP) tools enhanced real-time situational awareness by rapidly scanning global research articles, and news outlets to identify and synthesize emerging scientific insights (Jumper et al., 2021; Ren et al., 2020; Capponi et al., 2021). Collectively, these advances highlighted the transformative role of AI in outbreak response and set the stage for its broader integration into future pandemic preparedness strategies.

#### 7.5.2 Ebola virus

During the 2018–2020 Kivu outbreak in the Democratic Republic of the Congo, AI facilitated predictive risk mapping by integrating satellite imagery, climate data, and animal habitat distributions to identify spillover hotspots (Willmington et al., 2022; Pigott et al., 2014). Machine learning models were employed to differentiate between local transmission chains and novel viral introductions, thereby informing containment strategies and resource allocation. Furthermore, AI-driven phylodynamic frameworks provided critical insights into the evolutionary dynamics and geographic spread of the virus. Complementarily, network-based analyses of contact-tracing data identified key transmission pathways, which guided targeted vaccination campaigns in resource-constrained and conflict-affected settings.

#### 7.5.3 Mpox

The 2022 global Mpox outbreak highlighted AI’s potential in detecting atypical transmission. Machine learning analyses of genomic data confirmed sustained human-to-human spread and revealed hidden transmission chains beyond endemic regions (Gigante et al., 2022). Models integrating air travel and case data further predicted high-risk cities for importations, supporting proactive surveillance and public health messaging. In clinical diagnostics, CNN-based models distinguished Mpox from other skin lesions with accuracies ranging from 78 to 98.8% across multiple datasets and architectures (Chadaga et al., 2023), underscoring AI’s potential for rapid and reliable Mpox detection.

#### 7.5.4 Influenza

AI applications in influenza span routine forecasting and pandemic preparedness. In seasonal surveillance, U.S. CDC forecasts are augmented with models incorporating viral genomics, search engine data, and historical trends to predict epidemic timing and intensity (Reich et al., 2019). For pandemic risk assessment, AI evaluates avian influenza strains (e.g., H5N1, H7N9), predicting traits such as receptor binding specificity and antigenic drift to inform pre-pandemic vaccine libraries (Lou et al., 2024).

### 7.6 Advantages over traditional methods

The integration of AI into virology and epidemiology provides substantial advantages over conventional approaches, particularly in speed, scalability, and predictive power.

#### 7.6.1 Speed and automation

Traditional sequence analyses, such as BLAST searches and phylogenetic reconstructions, are computationally intensive and require manual curation. In contrast, trained AI models can process millions of sequences in hours, enabling real-time surveillance. Automated pipelines convert raw sequencing reads into variant calls and lineage assignments with minimal human intervention, allowing experts to focus on interpretation rather than data processing (Lee, 2023).

#### 7.6.2 Handling high-dimensional data

AI excels at integrating diverse datasets, including genomic sequences, protein structures, clinical outcomes, mobility patterns, and environmental variables, revealing complex, non-linear relationships that traditional methods cannot capture. Whereas logistic regression may detect a few predictors, machine learning models such as random forests or neural networks can uncover intricate interactions to predict patient severity or outbreak hotspots (Soenksen et al., 2022).

#### 7.6.3 Discovery of novel patterns

Unlike hypothesis-driven methods, AI can identify previously unrecognized patterns via unsupervised learning. This has enabled the discovery of novel CRISPR systems and microbial defense mechanisms (Doron et al., 2018). In virology, such approaches facilitate the detection of novel viral families and unconventional pathogenic mechanisms that might be overlooked by conventional analyses.

#### 7.6.4 Predictive accuracy and adaptability

Classical compartmental models, such as SEIR, rely on fixed parameters. AI-enhanced models continuously assimilate new data, adapting forecasts as outbreaks evolve. This adaptability improves short-term predictive accuracy, as demonstrated by ensemble models that consistently outperformed traditional methods during the COVID-19 pandemic (Cramer et al., 2022).

## 8 Challenges and proposed solutions

The integration of AI into viral metagenomics offers transformative potential for outbreak response, yet its journey toward widespread, reliable, and equitable implementation is constrained by significant challenges Critically examining these limitations is not intended to diminish the technology’s promise, but rather to provide a roadmap for guiding its continued evolution. This section highlights the central barriers, ranging from data availability and quality to model interpretability, to infrastructural and resource constraints—and aligns them with emerging research directions and technological innovations that seek to address these gaps.

### 8.1 Data scarcity and labeling bottlenecks

A foundational challenge is the “data requirements” of deep learning models, which require vast quantities of high-quality, accurately labeled sequences for training (Ren et al., 2020; Greener et al., 2022). The performance and generalizability of models are directly correlated with the volume and quality of their training data. However, the ground truth in virology is often elusive; labeling sequences as “viral” or assigning taxonomy requires slow, manual experimental validation or high-confidence homology, creating a fundamental data bottleneck (Edwards et al., 2016; Roux et al., 2019). As a result, viral sequence datasets remain orders of magnitude smaller than those used to train foundational models in other domains. Researchers often resort to data augmentation, semi-supervised learning, or transfer learning to partially overcome these limitations (Li et al., 2021; Santiago-Rodriguez and Hollister, 2022). While useful, these approaches are ultimately stopgap solutions; they cannot fully replace large-scale, high-fidelity, experimentally validated data.

More pernicious than sheer quantity is the profound taxonomic bias embedded within existing genomic databases. Public repositories like GenBank and RefSeq are overwhelmingly skewed toward viruses of established clinical and agricultural importance (e.g., influenza, HIV, SARS-CoV-2) (Nayfach et al., 2021; Tisza and Buck, 2021; Shkoporov et al., 2022; Schulz et al., 2020). In contrast, viruses from environmental niches, extreme ecosystems, and non-model organisms are severely underrepresented, creating a vast “viral dark matter” (Li et al., 2021; Santiago-Rodriguez and Hollister, 2022). This imbalance creates a “long-tail” distribution problem where DL models become highly accurate at recognizing common human pathogens but fail to identify novel or underrepresented viral families from under-sampled ecosystems, potentially delaying the response to a novel zoonotic spillover event (Nazer et al., 2023; Rampelli et al., 2020).

#### 8.1.1 Emerging solutions and research directions

A multi-faceted approach is being developed to combat data limitations:

1. Transfer learning and pre-trained models: Researchers are increasingly leveraging models pre-trained on massive, general-purpose protein or nucleotide sequence databases (e.g., models inspired by AlphaFold, DNABERT) (Jumper et al., 2021; Capponi et al., 2021). These models learn fundamental biological “grammar” and can be fine-tuned for specific viral classification tasks with much smaller, viral-specific datasets, thereby reducing the burden of data scarcity (Camargo et al., 2023).
2. Data augmentation and few-shot learning: Advanced techniques are being employed to artificially expand training datasets by generating realistic synthetic viral sequences (Ji et al., 2021). Furthermore, “few-shot learning” algorithms are being designed to learn effectively from a very small number of examples, which is critical for rare or novel viral families.
3. Global sequencing initiatives: Concerted efforts to systematically sequence diverse environments (e.g., the Global Virome Project, Earth Virome) are crucial for populating databases with novel viral sequences, thereby gradually correcting taxonomic biases and providing a more representative ground truth for model training (Rampelli et al., 2020).

### 8.2 The black box problem: interpretability and explainable AI

The predictive power of deep learning models is often tempered by their lack of interpretability, rendering them as inscrutable “black boxes” (Rudin, 2019; Greener et al., 2022). Although models may achieve high accuracy in distinguishing viral from host sequences, the underlying basis of their predictions often remains opaque. Identifying which specific nucleotides, motifs, or genomic structures drive a given decision is a persistent challenge. This lack of interpretability poses a critical barrier for virologists and public health officials, who need not only accurate classifications but also biologically meaningful and actionable insights to guide experimental validation and inform public health interventions (Barredo Arrieta et al., 2020; Samek et al., 2017).

#### 8.2.1 Emerging solutions and research directions

The field of Explainable AI (XAI) is becoming indispensable for building trust and transforming predictions into scientific discovery.

1. Saliency maps and gradient-based techniques: Methods like Grad-CAM can highlight the nucleotides in an input sequence that most strongly influence the model’s output, creating a “heatmap” of importance across the genome (Singh et al., 2016). For instance, when a model classifies a sequence as a coronavirus, a saliency map might pinpoint the receptor-binding domain, providing immediate, biologically plausible validation.
2. Feature attribution methods: Frameworks like SHAP (SHapley Additive exPlanations) quantify the contribution of each input feature to the final prediction (Lundberg and Lee, 2017). In viral host prediction, SHAP can reveal if a model is relying on codon usage bias or specific promoter sequences, thereby uncovering genomic signatures of co-evolution and generating testable hypotheses (Auslander et al., 2021).
3. XAI for discovery: Crucially, XAI extends beyond model debugging to enable biological insight. For instance, if an XAI model consistently highlights a non-structural protein gene in novel viruses associated with severe disease, it could point toward a previously uncharacterized virulence factor, guiding subsequent experimental research (Li et al., 2021; Choi et al., 2022). The development of domain-specific XAI tools is a critical research frontier for making AI a collaborative partner in virology.

### 8.3 Generalization and computational barriers

The development and training of state-of-the-art AI models require substantial GPU power and memory, creating a high financial and infrastructural barrier to entry for many academic and public health laboratories (Slavov, 2025). This is especially problematic in resource-limited settings where outbreak risks are often highest, threatening to create a new “AI divide” in global health security.

Furthermore, models trained on data from specific environments (e.g., human respiratory samples) often suffer from poor generalizability when applied to new contexts (e.g., seawater or animal vectors), a phenomenon known as overfitting (Li et al., 2023). A model that excels at identifying respiratory viruses in Illumina data from a US hospital may perform poorly on Nanopore data from bat samples in Southeast Asia, limiting its utility for proactive surveillance at the human-animal interface.

#### 8.3.1 Emerging solutions and research directions

Innovations in computational infrastructure and model design are beginning to mitigate these barriers:

1. Cloud-based platforms and pre-trained models: The growth of cloud computing allows researchers to access high-performance computing on demand (Le Piane et al., 2024). More importantly, the sharing of pre-trained models means that end-users can fine-tune existing powerful models for their specific tasks, bypassing the immense cost of training from scratch.
2. Federated learning: This distributed approach enables AI models to be trained collaboratively across multiple decentralized datasets (e.g., from hospitals in different countries) without the raw data ever leaving its local environment (Nazer et al., 2023); (Yurdem et al., 2024). This preserves data privacy and sovereignty while allowing for the creation of more robust and generalizable models from diverse data sources, directly addressing the generalizability challenge.
3. Model optimization and lightweight architectures: Active research into model compression, quantization, and the development of more efficient neural network architectures aims to create powerful yet lean models that can be deployed on less powerful hardware, including at the point-of-care with portable sequencers (Dantas et al., 2024).

### 8.4 Ethical considerations and governance

Beyond technical and computational barriers, the deployment of AI-powered metagenomics raises profound ethical questions that must be addressed to ensure responsible and equitable use. The capacity to sequence and analyze genetic material from any environment or patient with unprecedented speed creates new ethical dilemmas surrounding data privacy, bias, and the potential for unintended societal harm (Martinez-Martin and Magnus, 2019; Johnson et al., 2025). As NGS becomes increasingly integrated into clinical practice, the development of comprehensive, standardized regulations will be essential to effectively address its associated ethical challenges.

A primary concern is data privacy and consent. Clinical mNGS often sequences all nucleic acids in a sample, including the human host genome (Elbehiry and Abalkhail, 2025). This raises critical questions about patient autonomy and informed consent, as it is impossible to predict all pathogens that might be found. Furthermore, the integration of genomic data with clinical and mobility information in AI models creates rich datasets that are potentially re-identifiable, posing significant privacy risks if breached or misused.

Secondly, algorithmic bias and equity are major concerns (Joseph, 2025). As discussed in Section 6.1, models trained on biased data will perpetuate and potentially amplify these biases in their predictions. This can lead to systemic blind spots where pathogens circulating in under-surveilled regions are not detected, or where diagnostic AI tools perform poorly for certain populations. This could exacerbate global health inequities, directing resources and attention away from the most vulnerable communities.

Finally, the rapid identification of a novel pathogen with pandemic potential triggers complex questions about data sharing and dual-use risk. While rapid, open data sharing is crucial for a coordinated global response, it also creates a tension with national security and the risk of “dual use” research, where the same genomic information used to develop vaccines and diagnostics could theoretically be misused (Flores-Coronado et al., 2025). Establishing norms for the responsible communication of high-consequence findings to public health authorities without causing undue panic or stigma is a critical challenge.

#### 8.4.1 Emerging solutions and governance frameworks

Developing robust ethical and governance structures is as important as advancing the technology itself.

1. Strengthening consent frameworks: Moving toward dynamic or tiered consent models for metagenomic testing, alongside the development and use of robust data anonymization and secure, federated learning techniques, can help protect individual privacy (Elbehiry and Abalkhail, 2025).
2. Bias audits and equity-focused design: Implementing mandatory algorithmic bias audits and actively promoting the sequencing of diverse viromes are essential to build fair and representative models. The “fairness” of AI models must be a key performance metric alongside accuracy (Chen et al., 2023).
3. International governance and policy: The establishment of clear international guidelines and agreements on the timely sharing of pathogen genomic data, coupled with frameworks to manage dual-use concerns, is urgently needed. Organizations like the WHO are pivotal in facilitating this dialog to ensure that these powerful tools serve global public health interests equitably and responsibly (Johnson et al., 2025).

Despite these challenges, the integration of deep learning, especially transformer-based models, into viral metagenomics is reshaping the field. By moving from reliance on known references toward data-driven discovery, these approaches are essential for both rapid outbreak characterization and systematic exploration of the global virosphere. Overcoming data scarcity, reducing bias, improving interpretability, ensuring generalizability and ethical consideration will be critical for unlocking their full potential.

By confronting these challenges with the outlined strategies, the field is moving steadily toward the development of robust, interpretable, and globally accessible AI-powered metagenomic tools. The goal is not to create perfect models, but to build resilient systems where the combined strengths of mNGS and AI can be reliably leveraged in the high-stakes, time-sensitive environment of an emerging outbreak.

## 9 Future perspectives

The trajectory of AI and viral metagenomics points toward a fundamental shift in how we monitor, detect, and respond to infectious disease threats. The future lies not merely in refining individual technologies, but in their deep integration into proactive, intelligent, and equitable global health systems. This section outlines several concrete paradigms and key milestones that will define the next decade of outbreak response.

The concept of an “AI-first outbreak response” heralds a paradigm shift wherein AI technologies transition from being supportive tools to leading players in epidemiological investigations and response strategies (Kaur and Butt, 2025). Traditionally, outbreak management has been largely human-driven, relying heavily on expert input for hypothesis generation, prioritizing laboratory testing, and coordinating contact tracing (Kaur and Butt, 2025). In contrast, AI-based systems can now autonomously generate hypotheses about pathogen origins and transmission pathways by integrating sequencing data with epidemiological and mobility information (Ye et al., 2025). They can also accelerate pathogen detection through rapid sequence classification and predict transmission hotspots for targeted interventions (Srivastava et al., 2025). By adopting an AI-first approach, public health systems can become significantly more proactive, adaptive, and scalable, often anticipating outbreaks before they fully manifest (CR et al., 2023). This strategy stands to drastically reduce the delay between pathogen identification and public health action, mitigating outbreak impacts and saving lives on a global scale (Villanueva-Miranda et al., 2025). As AI capabilities continue to advance, this proactive framework is poised to become a cornerstone of modern infectious disease control (Kaur and Butt, 2025).

The integration of AI with mNGS is poised to transform outbreak response from a largely reactive process into a proactive, predictive, and globally coordinated system. Several developments will shape this future trajectory. First, the concept of a “Digital Immune System” envisions an AI-driven global surveillance network capable of continuously analyzing metagenomic, clinical, environmental, and mobility data streams (Afshinnekoo et al., 2015). Such systems would detect anomalies and novel genomic signatures with sufficient accuracy to trigger automated early warnings, potentially identifying outbreaks weeks before traditional reporting. Future developments should focus on creating AI systems that seamlessly mesh with existing public health surveillance infrastructures such as hospital reporting networks and environmental monitoring stations allowing for continuous, automated data ingestion and pathogen detection (Alwakeel, 2025; Kaur and Butt, 2025). This synergy will support the development of an early warning ecosystem capable of flagging viral emergence or mutations in real time, thereby allowing proactive measures to prevent large-scale spread (Villanueva-Miranda et al., 2025).

Second, advances in point-of-care metagenomics will enable rapid, field-deployable sequencing workflows, achieving “sample-to-answer” diagnostics in under 2 h (de Olazarra and Wang, 2023). Breakthroughs in portable hardware, lightweight AI algorithms, and curated “reference-on-a-chip” databases will make real-time sequencing feasible even in remote or resource-limited settings.

Third, federated learning frameworks will address data sovereignty and privacy concerns by enabling collaborative training of AI models without transferring raw genomic data across borders (Yurdem et al., 2024). This will foster equity, reduce taxonomic bias, and ensure that models remain generalizable across diverse populations and geographic regions. Cloud-based platforms combined with federated learning frameworks offer an innovative solution to the long-standing challenge of data sharing in pathogen surveillance (Aswini et al., 2025). Conventional centralized repositories often raise issues of privacy, sovereignty, and security, which discourage laboratories and countries from freely exchanging sensitive genomic data (Chourasia et al., 2024). Federated learning circumvents this by enabling AI models to be trained collaboratively across multiple decentralized datasets held by different organizations or countries, without the raw data ever leaving their local environments (Zwiers et al., 2024). This preserves patient confidentiality and national data ownership while harnessing the breadth of diverse, globally distributed datasets to produce more robust and generalizable AI models. Importantly, fostering such international collaboration is vital for pandemic preparedness and the detection of novel pathogens, as it enhances transparency, broadens surveillance capacity, and accelerates coordinated global responses (Zwiers et al., 2024; Calvino et al., 2024).

Fourth, the evolution of XAI will move AI beyond black-box predictions toward interpretable outputs that highlight key genomic features driving classification (Ali et al., 2023). This capability will enhance trust, accelerate biological discovery, and support evidence-based decision-making by public health authorities. The adoption of explainable AI will be critical for ensuring broad trust in AI-powered outbreak analytics among public health officials, researchers, and policymakers. Although AI can uncover intricate patterns and generate predictions from high-dimensional metagenomic data, its “black-box” nature risks undermining confidence if the reasoning behind outputs remains unclear (Giuste et al., 2023). To bridge this gap, explainability frameworks tailored for viral genomics and metagenomics will need to be developed, capable of providing domain-relevant insights into how models prioritize mutations, classify sequences, and generate risk assessments (Yagin et al., 2023). Such transparency not only enables experts to validate AI findings but also supports effective communication with non-specialist stakeholders, thereby strengthening decision-making and public understanding (Msomi et al., 2025; Abe et al., 2023). As these explanation tools evolve, they will empower epidemiologists to better interpret AI-driven alerts, identify false positives or novel biology, and ultimately improve the overall accuracy and credibility of outbreak investigations.

Together, these innovations point toward an AI-first outbreak response paradigm, where intelligent systems autonomously analyze data, generate hypotheses, predict transmission dynamics, and guide interventions, while human experts provide oversight and strategy. By making surveillance faster, more interpretable, and globally inclusive, AI-powered mNGS could become the cornerstone of a resilient defense against future pandemics.

## 10 Conclusion

The integration of artificial intelligence with viral metagenomics marks a paradigm shift in outbreak response, moving us from reactive diagnostics to proactive pandemic preparedness. AI directly addresses the core bottleneck of mNGS—data complexity—by enabling rapid pathogen identification, novel virus discovery beyond traditional methods, and predictive modeling of outbreaks. While challenges of data scarcity, model interpretability, and equitable access remain, emerging solutions like explainable AI and federated learning provide a clear path forward. This powerful synergy is forging a new “AI-first” frontier in global health, paving the way for intelligent surveillance systems capable of defending against future viral threats.
